# Comprehensive analysis of poly(A) tails in mouse testes and ovaries using Nanopore Direct RNA Sequencing

**DOI:** 10.1038/s41597-024-04226-8

**Published:** 2025-01-10

**Authors:** Agnieszka Czarnocka-Cieciura, Michał Brouze, Natalia Gumińska, Seweryn Mroczek, Olga Gewartowska, Paweł S. Krawczyk, Andrzej Dziembowski

**Affiliations:** 1 Laboratory of RNA Biology, International Institute of Molecular and Cell Biology, Warsaw, 02-109 Poland; 2 Institute of Genetics and Biotechnology, Faculty of Biology, University of Warsaw, Warsaw, 02-106 Poland; 3 Genome Engineering Facility, International Institute of Molecular and Cell Biology, Warsaw, 02-109 Poland; 4 Department of Embryology, Institute of Developmental Biology and Biomedical Sciences, Faculty of Biology, University of Warsaw, Warsaw, 02-096 Poland

**Keywords:** Oogenesis, Spermatogenesis, RNA sequencing, Bioinformatics

## Abstract

Gametogenesis is a process in which dysfunctions lead to infertility, a growing health and social problem worldwide. In both spermatogenesis and oogenesis, post-transcriptional gene expression regulation is crucial. Essentially, all mRNAs possess non-templated poly(A) tails, whose composition and dynamics (elongation, shortening, and modifications) determine the fate of mRNA. Moreover, gametogenesis, especially oogenesis, represents a unique instance of the complexity of poly(A) tails metabolism, with oocyte-specific waves of cytoplasmic polyadenylation. In this context, we provide a comprehensive transcriptomic dataset focusing on mRNA poly(A) tail composition and dynamics in murine testes and ovaries. It consists of RNA samples isolated from wild-type and transgenic mice lacking TENT5 polymerases, which can extend poly(A) tails in the cytoplasm. TENT5 deficiencies have serious consequences. For instance, the defect of TENT5D causes infertility in humans. The data described here are generated mainly using the Oxford Nanopore Direct RNA Sequencing (DRS) method, which provides ground-truth information about mRNA molecules, including poly(A) tail length and nucleotide content. For instance, we show the prevalence of uridilated tails in testicular mRNAs.

## Background & Summary

**Fig. 1:**
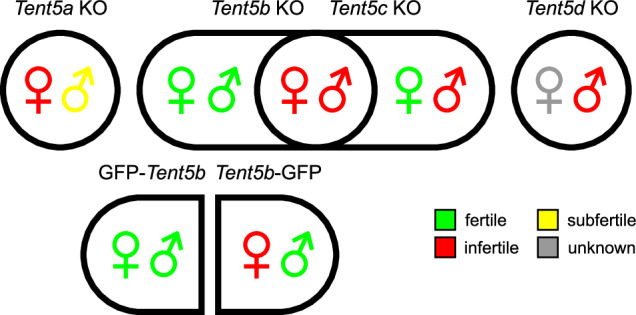
Schematic representation of the Tent5 phenotypes in mouse model. Summary of fertility status of males and females with all analyzed *Tent5* genes’ mutations. Subfertile category refers to significantly reduced fertility. Female *Tent5d* KO mice were unavailable for analyses due to *Tent5d* localisation on chromosome X and infertility of *Tent5d* KO males.

Development and maturation of germ cells constitute a multi-stage process governed by stringent gene expression and mRNA quality control mechanisms. During both oogenesis and spermatogenesis, diploid cells undergo meiotic division, leading to the generation of haploid gametes. Notably, the final stages of these processes occur when transcription is repressed, and regulation of gene expression relies primarily on the post-transcriptional mechanisms. In this context, the metabolism of mRNA poly(A) tails is critical. This poly(A) tail-dependent regulatory pathway involves the activity of various enzymes acting antagonistically at the 3′-end of the mRNA. Deadenylases operating both in the nucleus and the cytoplasm contribute to the shortening of poly(A) tails, while poly(A) polymerases extend them. Interestingly, cytoplasmic polyadenylation is particularly important for germ cell development. Mutations in *Tent5b*, *Tent5c*, and *Tent5d* genes encoding cytoplasmic poly(A) polymerases can affect both spermatogenesis and oogenesis (Fig. 1), as we uncovered in our recent work^1^. We described a notable wave of massive cytoplasmic polyadenylation in oogenesis and showed that double knock-out (KO) of *Tent5b/Tent5c* led to early arrest in oocyte development. Additionally, we detailed the effects of *Tent5c* and *Tent5d* KO on groups of transcripts critical to the progression of spermatogenesis. The importance of TENT5D for spermatogenesis is also highlighted by the fact that mutations in *TENT5D* lead to infertility in humans^1–4^. Growing evidence suggests that, in addition to the length of poly(A) tails, their nucleotide composition also affects mRNA stability^5,6^. Notably, the addition of uridines, termed uridylation, has been proven significant in gametogenesis. Conditional knock-outs of poly(U) polymerases *Tent3a* and *Tent3b* in mice have demonstrated their essential role, leading to infertility in both males and females^7,8^.

Despite the well-established importance of poly(A) tails, their methods of analysis, especially on the whole transcriptome scale, have remained imperfect and largely unchanged for decades. Poly(A) tails, like other homopolymer tracts, are prone to becoming unstable during amplification required in classical sequencing approaches^9,10^. This limitation can be addressed by using the amplification-independent Oxford Nanopore Direct RNA Sequencing (DRS) method. To date, DRS is the only technique that provides ground-truth information about poly(A) tails^9,11–16^. Furthermore, long DRS reads enable investigation of many other post-transcriptional gene expression regulatory mechanisms, such as alternative splicing and epitranscriptomic modifications. In nanopore sequencing, single-stranded nucleic acid molecules (RNA in the case of DRS) are passed through the protein pore due to applied voltage. This causes subsequent changes in the electric current, which are first recorded as a raw signal and then computationally converted to the corresponding nucleotide sequence. Based on the characteristic patterns (signatures) in the current intensity over time, poly(A) tails can be localized, and their lengths can be precisely estimated^9,12,13^. While the influence of poly(A) tail length on gene expression has been widely studied, the role of non-adenosine (non-A) residues within poly(A) tails remains poorly understood^5,6,9,17^. To fill this gap, we developed the Ninetails, a neural network-based algorithm, for the analysis of the nucleotide composition of poly(A) tails in DRS data (https://github.com/LRB-IIMCB/ninetails). Based on the properties of the raw signal of poly(A) tail region identified by other tools, our pipeline recognizes and quantifies non-As, such as cytosines, guanosines, and uridines, with high precision and recall.

**Fig. 2:**
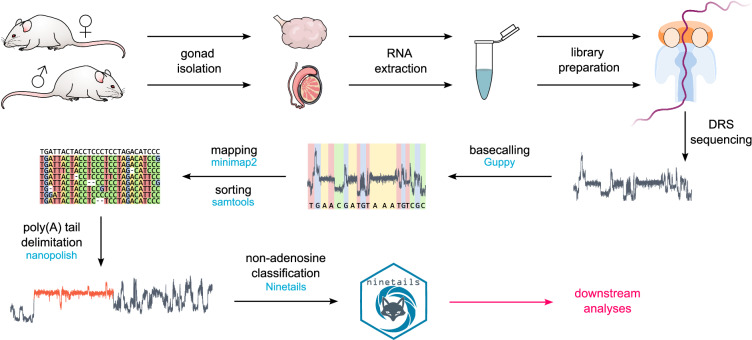
Schematic representation of the non-adenosine analysis workflow. Nanopore data were basecalled with Guppy, mapped with minimap2, sorted and filtered with samtools. The coordinates of poly(A) tails within the nanopore signals were delimited with Nanopolish. Finally, Ninetails was used to analyze the nucleotide composition of poly(A) tails.

**Fig. 3:**
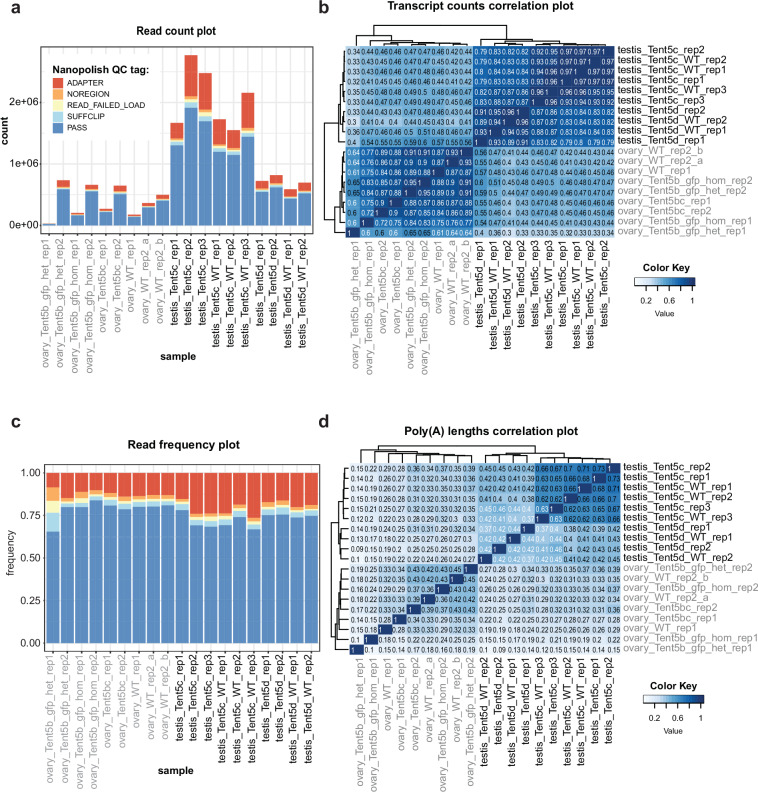
Quality of underlying DRS sequencing data. (**a**) Detailed classification of reads based on poly(A) tail length prediction quality tags provided by Nanopolish. The quality of the reads was independently summarized for each sample. (**b**) Correlation matrix for all DRS datasets. Spearman correlations were calculated for each pair of samples, taking into account mean transcript abundances (counts). (**c**) Frequency of reads labelled with quality tags calculated by Nanopolish for each sample. (**d**) Correlation matrix for all DRS datasets. Spearman correlations were calculated for each pair of samples, taking into account mean poly(A) tail lengths. The visualisations were produced with the NanoTail package.

Here, we present poly(A) tail composition DRS-based data derived from testes and ovaries of wild-type (WT) mice and animals with constitutive KO mutations of *Tent5* cytoplasmic poly(A) polymerases. These DRS datasets were initially described in our previous report focusing on poly(A) tail length profiling^1^. In this work, we augment them with the differential expression analysis and outputs of Ninetails software, providing a resource for detailed profiling of non-adenosine nucleotides in poly(A) tails of mRNAs from ovaries and testes without a cDNA proxy (Fig. 2). Importantly, our data enable orthogonal validation of previously published findings on the nucleotide composition of poly(A) tails in germline cells obtained with PacBio-based protocols^6,18^. The analysis of the non-A residues in poly(A) tails revealed an interesting observation that uridines are remarkably prevalent near the end of the tails in testes (Fig. 3), which ought to prompt further research. To gain more insight, we supplemented DRS with Illumina-based RNA-seq data obtained from a sorted cell population isolated from WT and *Tent5c* KO mice testes. These datasets supply information about the differential expression of genes involved in the maturation of male germ cells. Notably, a comparison of data on poly(A) tail composition with transcriptome changes occurring during differentiation revealed that in the transition from early postmeiotic cells (round spermatids) to later spermiogenesis stages (elongated spermatids), there is an overrepresentation of mRNAs with poly (A) tails rich in uridines among the downregulated mRNAs.

In general, the datasets provided will be useful to the broad scientific community interested in mammalian gametogenesis.

## Methods

### Experimental animal models

All material used for producing presented data was obtained from mice lines previously generated using CRISPR/Cas9 method in C57BL/6/Tar x CBA/Tar mixed background and described elsewhere^1,15,16^.

All experiments on animals were approved by the Local Ethical Committees at University of Warsaw, Faculty of Biology (approval numbers: 176/2026, 917/2019) and Warsaw University of Life Sciences, Faculty of Horticulture and Biotechnology (approval number: WAW2/049/2022)

For Illumina platform RNA sequencing, organs from following animals were used: 5-week-old *Tent5b*^+^^*/*^^+^*/Tent5c*^+^^*/*^,^+^ and *Tent5b*^*−/−*^*/Tent5c*^*−/−*^ females; 8-week-old *Tent5c*^+/+^ and *Tent5c*^*−/−*^ males.

For Direct RNA sequencing on MinION platform, organs from following animals were used: 30-days-old *Tent5b*^+^^*/*^^+^*/Tent5c*^+^^*/*^^+^, *Tent5b*^*−/−*^*/Tent5c*^*−/−*^, *Tent5b*^*wt/gfp*^ and *Tent5b*^*gfp/gfp*^ females; 8-week-old *Tent5c*^+^^*/*^^+^ and *Tent5c*^−^^*/*^^−^ males; 3-week-old *Tent5d*^*+/null*^ and *Tent5d*^*-/null*^ males.

### Germ cell isolation

Isolation of round and elongated spermatides from testes was performed as described previously^1^.

### RNA isolation

Total RNA from whole ovaries, testes and collected spermatides was isolated with TRI Reagent (Sigma) following manufacturer’s protocol. Tissues were homogenized in TRI Reagent using glass Dounce homogenizer while collected cell pellets were suspended in TRI Reagent and lysed by pipetting. DNA traces from final RNA samples were removed using TURBO DNA free kit (Thermo Fisher Scientific) before further experiments.

### Library preparation and sequencing

For spermatids and whole testes RNA sequencing, rRNAs were removed from total DNA-free RNA samples with Ribo-Zero Gold rRNA-removal Kit (Illumina). rRNA-free samples were cleaned up by the 3 M sodium acetate precipitation method. KAPA Stranded RNA-Seq Library Preparation Kit (KAPA Biosystems) was used for sequencing libraries preparation, with random primers used for cDNA synthesis step. At each step of the procedure, samples were cleaned up using AMPure XP Reagent magnetic beads. Quality and fragment size distribution in the libraries were determined by electrophoresis on Agilent 2100 Bioanalyzer (Agilent Technologies Inc.). Libraries for Direct RNA Sequencing (DRS) of ovarian and testicular RNA were prepared using the Direct RNA Sequencing kit (ONT, Cat# SQK-RNA002).

To improve the quality and efficiency of sequencing, we added 90–150 ng of *Saccharomyces cerevisiae* or *Schizosaccharomyces pombe* oligo(dT)-enriched mRNA to all samples as a carrier instead of RNA Control Strand supplied in the kit. This step was crucial for achieving desired sequencing stoichiometry without amplification, as the libraries obtained for ovaries and testes were low-input. Sequencing experiments were run on the MinION device loaded with Flow Cell Type R9.4.1 (ONT, Cat# FLO-MIN106D), controlled by the MinKNOW software (ONT).

### Preliminary sequencing data analysis

All raw DRS data^19–21^ were basecalled with Guppy 6.0.0 and mapped to the Gencode M26 reference transcript sequences^22^ using Minimap2 2.17^23^ with options -k 14 -ax map-ont –secondary = no. To remove supplementary alignments and reads mapping to reverse strand, we processed all datasets with samtools 1.9 (samtools view -b -F 2320)^24^ and discarded all unmapped reads. Next, poly(A) tail lengths were estimated using the Nanopolish 0.13.2^12^ polya function. The output of this software includes a quality tag indicating the validity of each tail estimation, which assigns reads to one of five categories. For most downstream analyses, we recommend using reads tagged as ‘PASS’ and ‘SUFFCLIP’ since these are technically correct and correspond to the reference. The exploratory data analysis was performed using NanoTail package^25^.

In addition to the data detailing the composition of poly(A) tails, we also present differential expression data^26^, which were not included in our previous work focused on differential adenylation. These results were obtained as described by us previously^1^.

Illumina datasets^27^ were mapped to the mouse GRCm39 genome using STAR^28^ with default settings. Aligned reads were summarized using featureCounts^29^ in the short-read mode (-p –O) and including only sequences covered by at least 50% of read (–fracOverlapFeature 0.5). Differential expression analysis was performed using DESeq2^30^.

### Poly(A) tail content analysis

We conducted an analysis of raw DRS data^19–21^ obtained from ovaries and testes to determine the occurrence of non-adenosines within poly(A) tails employing the Ninetails tool (see Code Availability) with the following parameters: qc = TRUE, pass_only = FALSE. The outputs of the Ninetails poly(A) surveillance pipeline in tsv format are provided^31,32^.

## Data Record

Here we present previously unpublished RNA-seq data has been deposited in Gene Expression Omnibus (GEO) repository under the accession number: GSE273264^27^ along with tables containing raw counts for each sample and results of differential expression analysis^27^. To reduce the background affecting statistical inference and data visualization (i.e., to alleviate potential high levels of variability in lowly expressed genes), log_2_ fold changes were shrunken.

The Table 1 with metadata of all RNA-seq and DRS samples has been uploaded to Figshare^33^. This table comprises 11 columns. The “sample_name” column contains the unique identifier of an independently sequenced sample, consistent across all published resources (e.g., in the corresponding differential expression analyses). The subsequent columns include the replicate number, species name (*Mus musculus*), organ (ovary/testis), and sequencing platform (ONT(DRS)/Illumina). These columns are particularly useful for clustering the data. The “project” and “accession” columns contain the unique identifiers of the projects and sequencing data records in the repositories. The “seq_data_link” column provides a direct hyperlink to each sequencing dataset (sample), while the “seq_source” column indicates whether and where the sequencing data has been previously published or if it is the original data presented herein. The last two columns, “nonA_data” and “diffexp_data”, indicate whether the corresponding nucleotide composition of poly(A) tails and differential expression data are provided in this work. In these columns, “Y” denotes the presence of the data, and “n” denotes its absence. The metadata table allows to seamlessly integrate the data from our previous work with the data record presented here.

**Table 1:** Metadata of deposited data record.

sample_name	replicate	species	organ	platform	project	accession	seq_data_link	seq_source	nonA_data	diffexp_data
ovary_Tent5bc_rep1	1	Mus musculus	ovary	ONT (DRS)	PRJEB63526	ERS15941056	https://www.ebi.ac.uk/ena/browser/view/ERS15941056	Brouze *et al*.^1^	Y	Y
ovary_Tent5bc_rep2	2	Mus musculus	ovary	ONT (DRS)	PRJEB63526	ERS15941057	https://www.ebi.ac.uk/ena/browser/view/ERS15941057	Brouze *et al*.^1^	Y	Y
ovary_WT_rep1	1	Mus musculus	ovary	ONT (DRS)	PRJEB63526	ERS16372230	https://www.ebi.ac.uk/ena/browser/view/ERS16372230	Brouze *et al*.^1^	Y	Y
ovary_WT_rep2_a	2	Mus musculus	ovary	ONT (DRS)	PRJEB63526	ERS15941059	https://www.ebi.ac.uk/ena/browser/view/ERS15941059	Brouze *et al*.^1^	Y	Y
ovary_WT_rep2_b	2	Mus musculus	ovary	ONT (DRS)	PRJEB46685	ERS5927539	https://www.ebi.ac.uk/ena/browser/view/ERS5927539	Brouze *et al*.^1^	Y	Y
ovary_Tent5b_gfp_het_rep1	1	Mus musculus	ovary	ONT (DRS)	PRJEB63526	ERS16372228	https://www.ebi.ac.uk/ena/browser/view/ERS16372228	Brouze *et al*.^1^	Y	Y
ovary_Tent5b_gfp_het_rep2	2	Mus musculus	ovary	ONT (DRS)	PRJEB63526	ERS16372248	https://www.ebi.ac.uk/ena/browser/view/ERS16372248	Brouze *et al*.^1^	Y	Y
ovary_Tent5b_gfp_hom_rep1	1	Mus musculus	ovary	ONT (DRS)	PRJEB63526	ERS15941063	https://www.ebi.ac.uk/ena/browser/view/ERS15941063	Brouze *et al*.^1^	Y	Y
ovary_Tent5b_gfp_hom_rep2	2	Mus musculus	ovary	ONT (DRS)	PRJEB63526	ERS16372252	https://www.ebi.ac.uk/ena/browser/view/ERS16372252	Brouze *et al*.^1^	Y	Y
testis_Tent5c_rep1	1	Mus musculus	testis	ONT (DRS)	PRJEB45063	ERS5846736	https://www.ebi.ac.uk/ena/browser/view/ERS5846736	Brouze *et al*.^1^	Y	Y
testis_Tent5c_rep2	2	Mus musculus	testis	ONT (DRS)	PRJEB45063	ERS5846735	https://www.ebi.ac.uk/ena/browser/view/ERS5846735	Brouze *et al*.^1^	Y	Y
testis_Tent5c_rep3	3	Mus musculus	testis	ONT (DRS)	PRJEB45063	ERS5846739	https://www.ebi.ac.uk/ena/browser/view/ERS5846739	Brouze *et al*.^1^	Y	Y
testis_Tent5c_WT_rep1	1	Mus musculus	testis	ONT (DRS)	PRJEB45063	ERS5846734	https://www.ebi.ac.uk/ena/browser/view/ERS5846734	Brouze *et al*.^1^	Y	Y
testis_Tent5c_WT_rep2	2	Mus musculus	testis	ONT (DRS)	PRJEB45063	ERS5846733	https://www.ebi.ac.uk/ena/browser/view/ERS5846733	Brouze *et al*.^1^	Y	Y
testis_Tent5c_WT_rep3	3	Mus musculus	testis	ONT (DRS)	PRJEB45063	ERS5846741	https://www.ebi.ac.uk/ena/browser/view/ERS5846741	Brouze *et al*.^1^	Y	Y
testis_Tent5d_rep1	1	Mus musculus	testis	ONT (DRS)	PRJEB45063	ERS5846730	https://www.ebi.ac.uk/ena/browser/view/ERS5846730	Brouze *et al*.^1^	Y	Y
testis_Tent5d_rep2	2	Mus musculus	testis	ONT (DRS)	PRJEB45063	ERS5846732	https://www.ebi.ac.uk/ena/browser/view/ERS5846732	Brouze *et al*.^1^	Y	Y
testis_Tent5d_WT_rep1	1	Mus musculus	testis	ONT (DRS)	PRJEB45063	ERS5465457	https://www.ebi.ac.uk/ena/browser/view/ERS5465457	Brouze *et al*.^1^	Y	Y
testis_Tent5d_WT_rep2	2	Mus musculus	testis	ONT (DRS)	PRJEB45063	ERS5465455	https://www.ebi.ac.uk/ena/browser/view/ERS5465455	Brouze *et al*.^1^	Y	Y
Mouse_testis_spermatids_WT_16	1	Mus musculus	testis	Illumina	PRJNA1141133	GSM8425435	https://www.ncbi.nlm.nih.gov/geo/query/acc.cgi?acc=GSM8425435	this work	n	Y
Mouse_testis_spermatids_TENT5C_KO_17	1	Mus musculus	testis	Illumina	PRJNA1141133	GSM8425436	https://www.ncbi.nlm.nih.gov/geo/query/acc.cgi?acc=GSM8425436	this work	n	Y
Mouse_testis_spermatids_WT_18	2	Mus musculus	testis	Illumina	PRJNA1141133	GSM8425437	https://www.ncbi.nlm.nih.gov/geo/query/acc.cgi?acc=GSM8425437	this work	n	Y
20170221_FamC_ES	1	Mus musculus	testis	Illumina	PRJNA1141133	GSM8425438	https://www.ncbi.nlm.nih.gov/geo/query/acc.cgi?acc=GSM8425438	this work	n	Y
20170221_FamC_RS	1	Mus musculus	testis	Illumina	PRJNA1141133	GSM8425439	https://www.ncbi.nlm.nih.gov/geo/query/acc.cgi?acc=GSM8425439	this work	n	Y
20170221_WT_ES	1	Mus musculus	testis	Illumina	PRJNA1141133	GSM8425440	https://www.ncbi.nlm.nih.gov/geo/query/acc.cgi?acc=GSM8425440	this work	n	Y
20170221_WT_RS	1	Mus musculus	testis	Illumina	PRJNA1141133	GSM8425441	https://www.ncbi.nlm.nih.gov/geo/query/acc.cgi?acc=GSM8425441	this work	n	Y
20170223_FamC_ES	2	Mus musculus	testis	Illumina	PRJNA1141133	GSM8425442	https://www.ncbi.nlm.nih.gov/geo/query/acc.cgi?acc=GSM8425442	this work	n	Y
20170223_FamC_RS	2	Mus musculus	testis	Illumina	PRJNA1141133	GSM8425443	https://www.ncbi.nlm.nih.gov/geo/query/acc.cgi?acc=GSM8425443	this work	n	Y
20170223_WT_ES	2	Mus musculus	testis	Illumina	PRJNA1141133	GSM8425444	https://www.ncbi.nlm.nih.gov/geo/query/acc.cgi?acc=GSM8425444	this work	n	Y
20170223_WT_RS	2	Mus musculus	testis	Illumina	PRJNA1141133	GSM8425445	https://www.ncbi.nlm.nih.gov/geo/query/acc.cgi?acc=GSM8425445	this work	n	Y
20170406_FamC_ES	3	Mus musculus	testis	Illumina	PRJNA1141133	GSM8425446	https://www.ncbi.nlm.nih.gov/geo/query/acc.cgi?acc=GSM8425446	this work	n	Y
20170406_FamC_RS	3	Mus musculus	testis	Illumina	PRJNA1141133	GSM8425447	https://www.ncbi.nlm.nih.gov/geo/query/acc.cgi?acc=GSM8425447	this work	n	Y
20170406_WT_ES	3	Mus musculus	testis	Illumina	PRJNA1141133	GSM8425448	https://www.ncbi.nlm.nih.gov/geo/query/acc.cgi?acc=GSM8425448	this work	n	Y
20170406_WT_RS	3	Mus musculus	testis	Illumina	PRJNA1141133	GSM8425449	https://www.ncbi.nlm.nih.gov/geo/query/acc.cgi?acc=GSM8425449	this work	n	Y
Mouse_testis_spermatids_TENT5C_KO_19	2	Mus musculus	testis	Illumina	PRJNA1141133	GSM8425450	https://www.ncbi.nlm.nih.gov/geo/query/acc.cgi?acc=GSM8425450	this work	n	Y

The Ninetails analysis was not performed on Illumina RNA-seq data, because the software is incompatible with the Illumina platform (requires ONT DRS data).

For both sequencing datasets differential expression analysis was performed. RNAseq data DESeq2 statistics was deposited Gene Expression Omnibus (GEO) repository^27^ and for DRS dataset in Figshare^26^.

The raw Ninetails results in tsv format are available on Figshare^31,32^. For each sample analyzed, Ninetails generates two output tables. Files with the suffix “class_data”^32^ contain the outcomes of read classification, with each row representing data for a single read. These files indicate whether the poly(A) tails of individual reads meet the quality criteria and whether they are decorated with non-adenosine residues or blank. Files with the suffix “residue_data”^31^ store information on specific non-adenosine occurrences. These files detail the type of non-adenosine found at a given position in a given tail and its length, with each row representing a single non-adenosine instance. A detailed explanation of the Ninetails output is available on Wiki (https://github.com/LRB-IIMCB/ninetails/wiki). The Ninetails output files are named according to the metadata table. These output data from Ninetails main analysis module (“check_tails” function) can then be transformed by post-processing functions (e.g., summarized, converted between wide and long format) and visualized with functions from the Ninetails graphics module. All these functionalities are covered in detail in the built-in help files for Ninetails and in the wiki, under Code Availability.

In tabular data, we avoid spaces and special characters, which facilitates further analysis using tools written in various programming languages.

## Technical Validation

For the 19 DRS source datasets^19–21^, the number of reads fluctuates between the samples (Fig. 3a), which is dependent on many determinants. One such factor is the amount of the starting material, since the DRS technology does not involve amplification. Notably, this does not affect the overall transcriptome overview, as the enrichment ratios of individual transcripts remain consistent between biological replicates (Fig. 3b). The distributions of read quality tags are similar across different replicates (Fig. 3c).

For the RNA-seq data, we performed correlation analysis, which revealed high homogeneity of the samples (strong positive correlation).

### Poly(A) tail length and composition

**Fig. 4:**
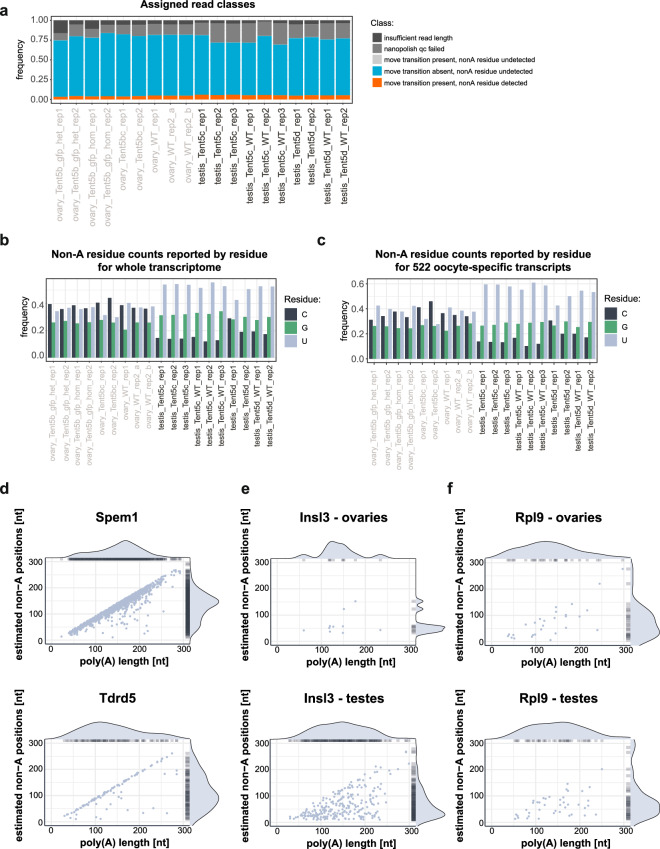
Distribution of non-adenosine nucleotides in poly(A) tails. (**a**) Detailed read classification made by Ninetails for poly(A) tail composition analysis. (**b**) Frequency of occurrence of cytidine/guanosine/uridine nucleotides in poly(A) tails calculated for whole transcriptome. Only reads classified by neural network (decorated, blank) were included in the analysis. (**c**) Frequency of occurrence of cytidine/guanosine/uridine nucleotides in poly(A) tails calculated for 522 oocyte-specific genes determined by Brouze *et al*.^1^. Only reads classified by neural network (decorated, blank) were included in analysis. (**d**) Localization of uridines in poly(A) tails of transcripts (*Spem1*, *Tdrd5*) involved in regulation of spermatogenesis. (**e**) Localization of uridines in poly(A) tails of transcript (*Insl3*) involved in regulation of spermatogenesis and oogenesis. (**f**) Localization of uridines in poly(A) tails of transcript (*Rpl9*) not involved in regulation of spermatogenesis and oogenesis. The visualisations were produced with the Ninetails software. Differential expression analysis performed on RNA-seq data.

To determine the poly(A) tail composition of 19 DRS datasets provided^19–21^, reads were categorized into five classes based on their quality and their adherence to the criteria required for identifying non-A residues in poly(A) tail. For subsequent analysis, only reads exhibiting a significant change between consecutive k-mers (so-called “move”) and displaying a characteristic anomaly in the nanopore raw signal were considered (Fig. 4a). Utilizing a neural network, the Ninetails program assigns the shape of the signal anomaly within a poly(A) tail to one of three categories, each representing anomalies corresponding to the presence of cytidine, guanosine, or uridine nucleotides. Next, we counted the frequency of each non-adenosine nucleotide in all experimental datasets (Fig. 4b) and for 522 oocyte-specific genes described in our previous work^1^ (Fig. 4c). The biological replicates were highly consistent both in terms of the size of the heterogeneous poly(A) tails fraction and in terms of their distribution among classes representing the respective non-adenosine nucleotides.

We observed no significant differences in the distribution of non-A nucleotides between the heterogeneous tails of oocyte-specific genes and all identified ovarian genes (Fig. 4b,c).

In samples obtained from murine testes, we identified an overrepresentation of uridines within the heterogeneous poly(A) tails (Fig. 4b). We further investigated the positions of non-adenosines within the poly(A) tails. As a result, we identified a subset of transcripts in which non-A residues predominantly occurred near the 3′-terminus of poly(A) tails in most reads (see Fig. 4d). These transcripts (e.g. *Spem1 and Tdrd5*) have been shown to be crucial to the progression of spermatogenesis^34–37^. Interestingly, the *Insl3* transcript, whose expression is necessary for the proper development of the urogenital tract and female fertility, has no enrichment in uridine at the end of poly(A) tails. The distribution of data points in the Fig. 4e panel is scattered, indicating a random insertion of uridines in the poly(A) tails. A similar pattern is seen for the *Rpl9* transcript (Fig. 4f), encoding a ubiquitous protein not involved in gamete differentiation. Thus, we suggest that the presence of non-adenosine nucleotides at the ends of their poly(A) tails is not a coincidence but rather a factor that regulates the expression of these mRNAs.

**Fig. 5:**
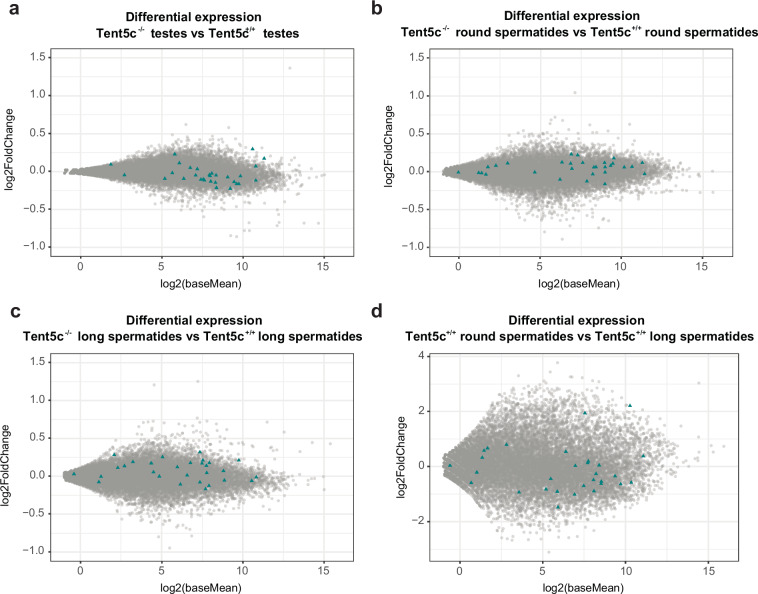
Differential expression of transcripts with enrichment in uridines at the 3′- end of poly(A) tail. (**a**) RNA-seq performed on testes isolated from Tent5c^*−/−*^ and Tent5c^+/+^ mice. Most transcripts with enrichment in uridines at the 3′-end of poly(A) tails, highlighted by triangles, are slightly downregulated in Tent5c^*−/−*^. Differential expression was calculated using DESeq2. (**b**) RNA-seq performed on round spermatids isolated from Tent5c^*−/−*^ and Tent5c^+/+^ mice. Most transcripts with enrichment in uridines at the 3′-end of poly(A) tails, highlighted by triangles, are slightly upregulated and highly expressed in Tent5c^*−/−*^. Differential expression was calculated using DESeq2. (**c**) RNA-seq performed on long spermatids isolated from Tent5c^*−/−*^ and Tent5c^+/+^ mice. Transcripts rich in uridines at the 3′-end of poly(A) tails are highlighted by triangles. Differential expression calculated using DESeq2. (**d**) RNA-seq performed on round spermatids and long spermatids isolated from Tent5c^+/+^ mice. Most transcripts with enrichment in uridines at the 3′-end of poly(A) tails, highlighted by triangles, are downregulated in long spermatids. Differential expression was calculated using DESeq2.

For testicular transcriptome analysis, RNA was isolated from *Tent5c*^−/−^ and *Tent5c*^+^^*/*^^+^ males. The testes of the *Tent5c*^−/−^ exhibit global changes in the expression of numerous genes. Although these differences may initially seem minor, the *Tent5c*^−/−^ mice demonstrate significant aberrations in the terminal stages of male gamete development^1^. Therefore, more substantial transcriptomic alterations are observed in spermatids. Additionally, the integration of data on the composition^26,31,32^ of poly(A) tails with changes in gene expression^27^ provides a novel perspective for the study of the regulatory mechanisms of spermatogenesis (Fig. 5).

## Data Availability

Information concerning the software used and its settings is included in the Methods section. The Ninetails tool is available at https://github.com/LRB-IIMCB/ninetails. The detailed user manual (wiki) for this software is available here: https://github.com/LRB-IIMCB/ninetails/wiki. Docker container and scripts used for neural network training with hyperparameter finetuning are provided here: https://github.com/LRB-IIMCB/ninetails_processing. The NanoTail software for exploratory analysis and visualization of poly(A) predictions produced by Nanopolish is available here: https://github.com/LRB-IIMCB/nanotail.
